# Influence of Running Surface Differences on Physiological and Biomechanical Responses During Specific Sports Loading

**DOI:** 10.3390/bioengineering12050534

**Published:** 2025-05-15

**Authors:** Zhiqiang Liang, Qi Shuo, Chuang Gao, Chang-Te Lin, Yufei Fang

**Affiliations:** 1 Faculty of Sports Science, Ningbo University, Ningbo 315211, China; liangzhiqiang@nbu.edu.cn (Z.L.); nbugaochuang@outlook.com (C.G.); 2 School of Sport and Health, Shandong Sport University, Jinan 250102, China; qishuo@sdpei.edu.cn; 3 State Key Laboratory of Advanced Marine Materials, Ningbo Institute of Materials Technology and Engineering (NIMTE), Chinese Academy of Sciences, Ningbo 315201, China; linzhengde@nimte.ac.cn; 4 Department of Rehabilitation Medicine, Ningbo No. 2 Hospital, Ningbo 315010, China

**Keywords:** running surface, joint kinetics, body composition, sports loading, muscle fatigue

## Abstract

The surface properties of the running surface have an effect on physiological and biomechanical responses to exercise, but their influence on body composition, blood pressure, and knee joint kinetics during controlled sports loading is less researched. This study compared the effects of treadmill running (TR) and overground running (OR) on acute physiological and biomechanical adaptation in ten male athletes aged between 23 and 26 years old following a 30 min protocol at 75% VO_2_max. Pre- and post-running body composition (fat volume, protein content, and fluid distribution), blood pressure, and knee joint kinetics (total work of muscle extensors—TWMEs) were assessed using bioelectrical impedance analysis, blood pressure monitor, and isokinetic dynamometry. The results indicated that TR led to highly significant reductions in protein content with a considerable accumulation of intracellular fluid. At the same time, TR reduced knee TWME by 27.4%, and OR elevated TWME by 5.6%. No significant differences in blood pressure were observed. These findings highlight surface-specific metabolic stress and biomechanical loading patterns and show that TR augments catabolic responses and knee joint strain despite equivalent external workloads.

## 1. Introduction

Running has emerged as one of the most prevalent aerobic exercises due to its well-documented health benefits, including improved cardiovascular fitness, metabolic control, and neuroprotection against age-related cognitive decline [1,2,3]. While physiological advantages of running are well-established, emerging evidence suggests that the biomechanical and metabolic burden of running may vary substantially with the training surface under some sports loading conditions [4]. Of many running conditions, treadmill running (TR) and overground running (OR) are two modalities that are distinct from one another, yet their comparative impacts on body composition and knee joint kinetics during controlled exercise intensity remain controversial.

Previous biomechanical studies have mostly been focused on kinematic differences between TR and OR, e.g., joint kinematic parameters, ground reaction forces, electromyography, and others [5,6,7]. As would be predicted from biomechanical performance between the two running surfaces, metabolic analysis indicates that TR has higher energy costs at the same kinematic [8]. These differences imply that surface differences may initiate varying physiological adaptation and mechanical loading differences. But acute body composition changes and knee joint kinetics during controlled running, although pertinent to the risk of injury and the optimization of performance, are infrequently assessed [7]. This lack constrains the understanding of how various running surfaces affect direct physiological and knee biomechanical responses, an essential factor for personalizing exercise prescriptions.

Comparing body composition on both running surfaces would provide significant information regarding exercise-induced physiological adaptation, including fluid redistributions, protein turnover, and fat metabolism [9,10]. Through the integration of body composition assessments with knee-specific kinetic analyses, this study provides improved mechanistic understanding of how varying running surface impacts physiological and biomechanical responses under controlled sports loading, informing training efficacy and physiological strain. The primary aim of the present investigation was to assess the acute response of TR and OR on the body composition, blood pressure, and knee joint kinematics during a 30 min run at standardized intensity corresponding to moderate-to-vigorous sporting loading. TR was expected to induce greater metabolic stress compared with OR; TR would exaggerate the knee joint loading, thereby leading to extreme decreases in the work of muscle extensors owing to muscle fatigue.

## 2. Methods

### 2.1. Subjects

Ten elite male running athletes (age: 24.5 ± 1.29 years; height: 177.3 ± 2.62 cm; weight: 68.4 ± 4.04 kg; training volume: 153.6 ± 8.37 km/week; training years: 8.6 ± 1.34 years) with no history of lower-limb injuries or cardiovascular diseases were recruited (Table 1). Subjects were required to maintain their habitual physical activity levels and avoid strenuous exercise 48 h prior to testing. The exclusion of female subjects aimed to control for potential hormonal influences on body composition and joint kinetics 19. All subjects provided written informed consent, and the study protocol was approved by the Research Ethics Committee of Grand Health.

### 2.2. Experimental Design

**Figure 1:**
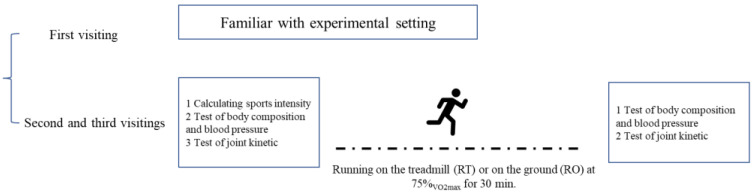
The procedure test of running at 75% VO_2_max.

A randomized crossover design was implemented to compare the effects of TR and OR under standardized sports loading conditions (Figure 1). Each subject completed three laboratory visits: once to get familiar with the experimental setting, and twice for 30 min running at 75% of the maximum oxygen uptake (75% VO_2_max) on either RT or OR, with a minimum 72 h washout period between sessions to eliminate residual fatigue. During the running session, tests of body composition, blood pressure, and knee joint isokinetic were completed before and after running, obtaining information on physiological and biomechanical performance.

### 2.3. Standardized Sports Loading Protocol

Subjects had to complete TR (H/P/cosmos, Nußdorf, Germany) and OR for 30 min. The target intensity (75% VO_2_max) was determined using the Karvonen equation [11]. Heart rate was continuously monitored using a Polar H10 chest strap (Polar Electro, Helsinki, Finland) during running. Running speed on TR was adjusted incrementally until the target heart rate stabilized (±3 bpm) within 5 min. Overground running speed was matched using a GPS-enabled smartwatch (Garmin Forerunner 945, Olathe, KS, USA). Once they did, they had to continue running for 30 min at the given sports intensity on TR and OR, which was tracked and regulated by a Polar belt.

### 2.4. Body Composition Test, Blood Pressure, and Joint Kinetics Test

Pre- and post-exercise measurements of body composition were conducted using a bioelectrical impedance analyzer (InBody 770, Seoul, Republic of Korea). Parameters included fat-free weight (FFW), muscle volume (MV), body moisture (BM), extracellular fluid (EF), protein (P), bone density (BD), fat volume (FV), body fat rate (BFR), body mass index (BMI), systolic pressure (SP), and diastolic pressure (DP). Blood pressure (systolic/diastolic) was measured using an automated monitor (HEM-7112, Hangzhou, China).

Isokinetic knee extensor strength was assessed using a Contrex MJ dynamometer (Contrex, Siegburg, Germany) at an angular velocity of 120°/s. During the measurement process, subjects maintained a seated posture, and the initial angle of the dominant knee joint was set to 90 degrees. To evaluate the effect of running surface on joint kinetic performance, the total work of muscle extensors (TWME) was calculated as the cumulative work output (in watts) over 10 maximal contractions.

### 2.5. Statistical Analysis

Data are presented as mean ± standard deviation (SD). Normality was verified using the Shapiro–Wilk test. Paired *t*-tests compared differences in body composition and knee kinetics between TR and OR. Statistical significance was set at *p* < 0.05. Analyses were performed using SPSS 26.0 (IBM, Armonk, NY, USA).

## 3. Results

### 3.1. Running Performance Under Specific Sports Loading

All subjects completed the 30 min running protocol at 75% VO_2_max on both TR and OR. Despite the matched exercise intensity, the running speed during OR (13.69 ± 0.28 km/h) was marginally faster than during TR (13.50 ± 0.24 km/h), although this difference was not statistically significant (*p* = 0.732). Similarly, total distance covered on OR (6.85 ± 1.41 km) exceeded TR (6.72 ± 1.32 km), but without significance (*p* = 0.681).

### 3.2. Surface-Specific Differences in Body Composition

Significant differences between TR and OR in body composition were observed (Table 2). After running, TR elicited a significant increase in MV, BM, IF, and EI compared to OR (*p* < 0.05) and elicited a significant decrease in P than OR (*p* < 0.05). There was no discernible difference between TR and OR in terms of weight, BMI, FV, BD, BFR, SP, and DP (*p* > 0.05).

### 3.3. Physiological Strain and Recovery

**Figure 2:**
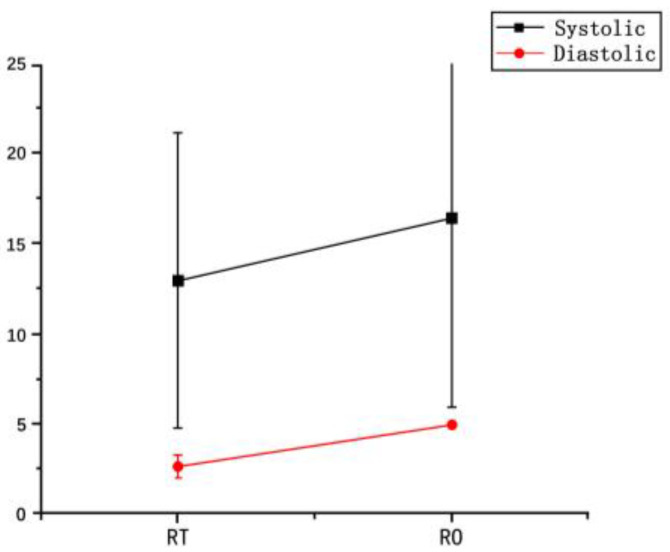
The variation in blood pressure between pre-/post-running.

Blood pressure responses were comparable between TR and OR (Table 2, Figure 2), no significant differences in systolic pressure were found (*p* > 0.05).

### 3.4. Knee Joint Kinetic Responses

The knee TWME between TR and OR showed a significant difference (*p* = 0.002); TR induced a 27.4% decline in TWME post-exercise, whereas OR showed a 5.6% increase.

## 4. Discussion

The present study investigated the acute effects of TR and OR on physiological and biomechanical responses, via monitoring the body composition, blood pressure, and knee joint kinetics before and after 30 min running at 75% VO_2_max. The findings support the hypotheses: TR resulted in much greater reductions in P than did OR, consistent with its larger metabolic demand. TR caused a significant decrement in TWME at the knee joint, suggesting surface-specific fatigue mechanisms. These results highlight the complex interaction between surfaces of running, metabolic adaptation, and biomechanical loading with respect to exercise prescription and injury prevention.

The significant reduction in P after TR is consistent with previous reports linking treadmill running with greater muscle catabolism [12]. As expected, TR’s higher cost of energy would have accelerated glycogen depletion to demand energy utilization from protein reserves [13,14]. This metabolic shift could explain the greater loss of protein during TR compared with OR. Similarly, the decline in FV during TR provides evidence favoring the thesis that higher mechanical stresses on treadmills induce higher lipid mobilization. Milanese et al. [15] had earlier shown that fat oxidation is positively related to exercise intensity, and our results apply this concept to surface-dependent loading. Of particular interest, the acute fluid redistribution (intracellular fluid increase in TR) also indicates adaptive processes to repeated impact forces since extracellular-to-intracellular fluid shifts have been shown to mitigate cellular stress during high-load exercise [16,17]. This is because the temporary loss of dissolved substances in body fluids alters water redistribution across cell membranes, and when the lost fluid is hypotonic relative to plasma, an osmotic gradient forms, leading to the distribution of water loss between the intracellular fluid and extracellular fluid spaces [18].

The TWME trends diverging between TR and OR are of the utmost importance for understanding surface-specific fatigue mechanisms. OR elicited a post-exercise increase in knee extensor work, while TR elicited a 27.4% decrease in TWME, consistent with cumulative muscle fatigue. This may be because treadmill running has limited biomechanics that limit stride variation and encourage repetitive quadriceps mechanism strain [19,20]. Reduced eccentric loading of TR’s belt-driven movement may further decrease muscle activation efficiency, worsening fatigability [21]. These findings are consistent with Rivera et al. [22] and Cronin et al. [23], who linked reduced muscle power output to monotonous loading patterns. Clinically, the reduction in TWME emphasizes caution against the overuse of high-intensity treadmill prescription as chronic fatigue can undermine joint stability and raise injury risk [24,25].

Unexpectedly, no significant differences in running distance or speed were found between TR and OR, with higher metabolic cost in TR. This paradox may be explained by psychological factors (e.g., subjective effort during TR) or biomechanical compensation (e.g., reduced vertical oscillation) that mask true energy expenditure differences [26,27,28]. This difference may be an artifact of our focus on acute responses rather than chronic adaptations, emphasizing the need for longitudinal studies.

This study has several limitations that must be considered in subsequent studies. Firstly, the use of only male subjects precludes generalization to female athletes, whose body composition and fatigue responses could be dissimilar for hormonal and morphological reasons [29,30]. Previous studies have reported that in men and women who train at the same intensity, frequency, and duration, there seems to be no difference in the relative increase in VO_2_max. However, hormonal factors lead to a higher initial level of high-density lipoprotein in women, which results in a smaller change in the total cholesterol-to-high-density lipoprotein ratio caused by aerobic training in women compared to men [31]. This finding has certain limitations when used to explain female running performance on different running surfaces.

Secondly, this study measured only 10 men, which constitutes a relatively small sample size. This may hinder the ability to comprehensively represent the target male population, thereby potentially affecting the generalizability and reliability of the research findings to some extent. Thirdly, the absence of direct measures of metabolism (e.g., analysis of gas exchange) limits mechanistic interpretations of energy substrate utilization. Subsequent research must involve multimodal measurements (e.g., electromyography and blood lactate) to delineate biomechanical and metabolic contributions. Finally, the acute nature of the protocol may fail to reflect long-term adaptations; longitudinal designs must be used to determine chronic effects.

Despite these limitations, the findings in this study offer practical implications for actionable interventions: (1) Training Optimization: TR’s higher metabolic stress can be used to the benefit of athletes who desire rapid fat loss or metabolic conditioning, while OR’s lower knee joint fatigue may be better suited for endurance training. (2) Rehabilitation: Clinicians must manage knee extensor fatigue in treadmill-rehabilitating patients, perhaps altering surfaces to prevent overloading. (3) Individualized Prescription: Individual biomechanical profiles (e.g., stride variability and joint mobility) should guide surface selection to balance efficacy and injury risk.

## 5. Conclusions

The findings of this study demonstrate that differential effects of running surfaces on acute physiological and biomechanical reactions exist under standardized sports loading. TR induced greater metabolic stress, as evidenced by reductions in fat volume and protein content, along with intracellular fluid redistribution, which presumably reflect adaptive changes to repetitive mechanical loading. Conversely, TR induced notable knee joint fatigue (27.4% reduction in TWME), which was different from OR, which enhanced the efficiency of knee extensors. These findings suggest that TR imposes more catabolic stress and biomechanical loading on the knee, which may compromise joint stability with prolonged use.

## Figures and Tables

**Table 1:** The information on male subjects and sports intensity.

MHR (Times)	RHR (Times)	Sports Intensity		
		Up Limit (Times)	Target HR (Times)	Down Limit (Times)
195.5 ± 1.29	61.3 ± 0.58	166.67 ± 1.15	160.67 ± 1.15	154.67 ± 1.53

Note: MHR was maximum heart rate; RHR was resting heart rate; HR was heart rate. All data are expressed as mean ± standard deviation.

**Table 2:** Changes in body composition and blood pressure between TR and OR.

Index	TR		OR		Difference		*p*
	Pre	Post	Pre	Post	TR	OR	
Weight (kg)	67.38 ± 3.63	66.48 ± 4.04	68.43 ± 4.04	67.57 ± 4.14	0.90 ± 0.74	0.87 ± 0.35	0.946
Fat-free weight (kg)	58.20 ± 1.69	59.28 ± 1.83	59.37 ± 1.62	59.5 ± 1.75	−1.08 ± 0.24	−0.33 ± 0.035	0.008 *
Muscle volume (kg)	55.20 ± 1.61	56.20 ± 1.75	56.33 ± 1.56	56.43 ± 1.70	−1.00 ± 0.24	−0.10 ± 0.30	0.014 *
Body moisture (kg)	39.95 ± 1.62	42.05 ± 1.87	41.57 ± 1.07	42.23 ± 1.44	−2.10 ± 0.37	−0.67 ± 0.42	0.005 *
Intracellular fluid (kg)	25.28 ± 1.16	27.05 ± 1.38	26.43 ± 0.74	27.10 ± 1.04	−1.78 ± 0.33	−0.67 ± 0.32	0.008 *
Extracellular fluid (kg)	14.75 ± 0.47	15.03 ± 0.53	15.13 ± 0.35	15.13 ± 0.40	−0.28 ± 0.09	0.00 ± 0.10	0.015 *
Protein (kg)	15.28 ± 0.80	14.23 ± 0.73	14.87 ± 0.64	14.26 ± 0.60	1.05 ± 0.24	0.60 ± 0.17	0.041 *
Bone density (kg)	3.00 ± 0.08	3.08 ± 0.10	3.03 ± 0.06	3.07 ± 0.06	−0.08 ± 0.05	−0.33 ± 0.06	0.352
Fat volume (kg)	9.12 ± 2.36	7.23 ± 2.77	9.10 ± 2.63	8.10 ± 2.62	1.95 ± 0.72	1.00 ± 0.10	0.077
Body fat rate (%)	13.50 ± 2.69	10.68 ± 3.41	13.13 ± 2.99	11.80 ± 3.08	2.83 ± 1.09	1.33 ± 0.15	0.072
Body mass index (m^2^/Kg)	21.45 ± 1.48	21.18 ± 1.54	22.07 ± 1.17	21.80 ± 1.18	0.28 ± 0.28	0.27 ± 0.15	0.965
Systolic pressure(KPa)	122.67 ± 7.51	135.67 ± 2.08	127.50 ± 26.16	144.00 ± 15.56	13.00 ± 8.19	16.50 ± 10.61	0.700
Diastolic pressure(KPa)	69.33 ± 2.89	71.00 ± 2.65	66.00 ± 12.72	61.00 ± 11.31	2.67 ± 0.58	5.00 ± 1.42	0.722

* Indicates the significant difference between TR and OR at 75% VO_2_max. All data are expressed as mean ± standard deviation.

## Data Availability

The original contributions presented in the study are included in the article, further inquiries can be directed to the corresponding author.

## Abbreviations

The following abbreviations are used in this manuscript:

TR	Treadmill running
OR	Overground running
FFW	Fat-free weight
MV	Muscle volume
BM	Body moisture
EF	Extracellular fluid
P	Protein
BD	Bone density
FV	Fat volume
BFR	Body fat rate
BMI	Body mass index
TWME	Total work of muscle extensor
